# Multivariate Modeling for Spatio-Temporal Radon Flux Predictions

**DOI:** 10.3390/e25071104

**Published:** 2023-07-24

**Authors:** Sandra De Iaco, Claudia Cappello, Antonella Congedi, Monica Palma

**Affiliations:** 1 National Future Center of Biodiversity, 90133 Palermo, Italy; 2 DES-Sect. of Mathematics and Statistics, University of Salento, 73100 Lecce, Italy; 3 National Center of High Performance Computing, Big Data and Quantum Computing, 40121 Bologna, Italy

**Keywords:** multiple correlation, space–time coregionalization model, space–time prediction, cokriging

## Abstract

Nowadays, various fields in environmental sciences require the availability of appropriate techniques to exploit the information given by multivariate spatial or spatio-temporal observations. In particular, radon flux data which are of high interest to monitor greenhouse gas emissions and to assess human exposure to indoor radon are determined by the deposit of uranium and radio (precursor elements). Furthermore, they are also affected by various atmospheric variables, such as humidity, temperature, precipitation and evapotranspiration. To this aim, a significant role can be recognized to the tools of multivariate geostatistics which supports the modeling and prediction of variables under study. In this paper, the spatio-temporal distribution of radon flux densities over the Veneto Region (Italy) and its estimation at unsampled points in space and time are discussed. In particular, the spatio-temporal linear coregionalization model is identified on the basis of the joint diagonalization of the empirical covariance matrices evaluated at different spatio-temporal lags and is used to produce predicted radon flux maps for different months. Probability maps, that the radon flux density in the upcoming months is greater than three historical statistics, are then built. This might be of interest especially in summer months when the risk of radon exhalation is higher. Moreover, a comparison with respect to alternative models in the univariate and multivariate context is provided.

## 1. Introduction

Radon is a colorless, odorless, tasteless, inert radioactive gas, that is derived from the decay of uranium, which is a radioactive element found in small quantities in all sediments and rocks. According to the IARC (International Agency for Research on Cancer) and the WHO (World Health Organization), radon pollution represents the second leading cause of lung cancer after smoking. Since radon is present in the depths of the Earth in gaseous phase, it reaches the surface after interacting with other natural elements, such as uranium and radio (precursor elements). Moreover, various atmospheric variables, such as humidity, temperature and precipitation [1,2], also affect the transport of radon on the surface. In the literature, different variables related to radon migration from the soil to the atmosphere were used and various methods based on the spatial regression techniques (i.e., Geographical Weighted Regression, Empirical Bayesian Regression Kriging, Machine Learning and Forecast Regression) were proposed [3,4,5,6,7]. However, these contributions focus on the analysis of spatial radon distribution, while disregarding the opportunity to propose a spatio-temporal modeling where the spatial and temporal dimensions of the investigated phenomenon, as well as their possible interactions are considered. In this context, geostatistics can offer appreciable techniques and tools to face estimation problems in space and time, not only for the univariate case, but also for the multivariate one, which represents an innovative approach for the study of radon data. For this last, it is crucial to estimate the spatio-temporal multiple covariance function and define an apt multivariate correlation model which can ensure reliable inference on the radon flux variable. Indeed, efforts are focused on the estimation and modeling of the matrix-valued covariance function, which explains the direct and cross linear dependence in space or in space–time among the variables.

Besides models for multivariate spatial data which were extensively explored [8,9,10,11], various contributions can be found in the literature regarding multivariate spatio-temporal data modeling starting from the early nineties [12,13,14,15,16,17,18]. However, the linear coregionalization model (LCM) developed in space and in space–time (ST-LCM) is sufficiently flexible computationally speaking to be applied extensively for a large variety of scientific fields [9,19,20,21,22,23]. Li et al. [24] proposed a methodology to evaluate the appropriateness of several common assumptions, such as symmetry, separability and linear model of coregionalization, on multivariate covariance functions in the spatio-temporal context, while Choi et al. [13] proposed an ST-LCM where the multivariate spatio-temporal process was expressed as a linear combination of independent Gaussian processes in space–time with mean zero and a separable spatio-temporal covariance. Apanasovich & Genton [25] considered some solutions to the symmetry problem; moreover, they proposed a class of cross-covariance functions for multivariate random fields based on the work of Gneiting [26]. The maximum likelihood estimation of heterotopic spatio-temporal models with spatial *LCM* components and temporal dynamics was developed by Fassó and Finazzi [27]. A *GSLib* [28] routine for cokriging was properly modified in De Iaco et al. [29] to incorporate the ST-LCM, previously developed [15] using the generalized product–sum variogram model. In [30,31,32], an automatic procedure for fitting the ST-LCM was presented and some computational aspects, analytically described by a main flow-chart, were discussed. Simultaneous diagonalization of the sample matrix variograms or the sample covariance matrices was used to isolate the basic components of an ST-LCM and it has been illustrated how nearly simultaneous diagonalization of the covariance matrices simplifies their modeling.

This paper aims at presenting a spatio-temporal multivariate geostatistical modeling approach, based on joint diagonalization of the empirical covariance matrices evaluated at different spatio-temporal lags. Thus, the possibility to consider a reduced number of uncorrelated variables (lower than the number of observed variables) and to separately model the spatio-temporal evolution of these uncorrelated components represents a substantial simplification in multivariate modeling. A space–time linear coregionalization model (ST-LCM) with proper parametric models for the latent components was fitted to the matrix-valued covariance function estimated for the radon flux and three relevant atmospheric variables, which include evapotranspiration, minimum humidity and mean temperature. The analysis highlighted how to identify the space–time components and choose the corresponding model by evaluating some characteristics of the components, such as separability and type of non-separability. Apart from the practical importance of this study in the specific field of application, it empirically proves the flexibility of modeling the matrix-valued covariance structure using the ST-LCM when more than two or three variables are involved. This is made possible through an approach based on the joint diagonalization of sample covariance matrices at different lags. Indeed, it enables analysts to overcome the complexity of fitting the ST-LCM, particularly when the number of variables to be analyzed increases, as it does not require modeling all direct and cross-covariance functions. Furthermore, through the aforementioned procedure, analysts can easily identify the basic components of the ST-LCM and model each component according to its empirical characteristics, which may necessitate the use of different classes of covariance models featuring various types of non-separability [33].

In the following, after an introduction of the theoretical framework of the multivariate spatio-temporal random function and its features (Section 2), the ST-LCM, its assumptions and appropriate statistical tests are presented (Section 3), then techniques for prediction and risk assessment maps are introduced (Section 4). Finally, the spatio-temporal multivariate exploratory analysis concerning radon flux and three atmospheric variables (temperature, humidity and evapotranspiration) in the Veneto Region (Italy), and the subsequent modeling step are provided (Section 5 and Section 6). Predictions of the primary variable (radon flux) are obtained through spatio-temporal cokriging (Section 7) for some future months. Then, risk maps showing the probability that the radon flux in a summer month exceeds the value of some chosen statistics (25th percentile, average, median), computed on the corresponding historical data, are produced (Section 8). The choice of summer month is justified by the need to investigate the risk of radon exhalation when warmer climatic conditions in the study area favor the increase of radon flux.

It is worth pointing out that the prediction results of this multivariate study might be of interest for their reflections in public health and for planning consequent remediation strategies.

## 2. Multivariate Spatio-Temporal Random Function

Let $Z\left(u\right)={\left[Z_{1}\left(u\right),\ldots ,Z_{p}\left(u\right)\right]}^{T},$ be a vector of *p* spatio-temporal random functions (*STRF*) defined on the domain $D\times T\subseteq R^{d+1}$, with (d≤3), then

$$\{Z\left(u\right),\;u=\left(s,t\right)\in D\times T\subseteq R^{d+1}\},$$

represents a multivariate spatio-temporal random function (*MSTRF*), where $s=(s_{1},\ldots ,s_{d})$ are the coordinates of the spatial domain $D\subseteq R^{d}$ and *t* the coordinate of the temporal domain T⊆R.

Afterwards, the *MSTRF* will be denoted by Z and its components by $Z_{i}$. The *p* *STRF* $Z_{i},\;i=1,\ldots ,p,$ are the *components* of Z and they are associated to the spatio-temporal variables under study; these components are called *coregionalized variables* [34]. The observations $z_{i}\left(u_{\alpha }\right),\;i=1,\ldots ,p,\;\alpha =1,\ldots ,N_{i},$ of the *p* variables $Z_{i}$, at the points $u_{\alpha }\in D\times T$, are considered a finite realization of an *MSTRF* Z and $N_{i}$ is the number of spatio-temporal points for the variable $Z_{i}$.

An *MSTRF* Z, with *p* components, is *second-order stationary* if:

- for any *STRF* $Z_{i},\;i,\ldots ,p,$
  (1) $$E\left[Z_{i}\left(u\right)\right]=m_{i},\;u\in D\times T,\;i=1,\ldots ,p;$$
- for any pair of *STRF* $Z_{i}$ and $Z_{j},\;i,j=1,\ldots ,p,$ the cross-covariance $C_{ij}$ depends only on the spatio-temporal separation vector $h=(h_{s},h_{t})$ between the points u and u+h:
  (2) $$C_{ij}\left(h\right)=E\left[\left(Z_{i}\left(u+h\right)-m_{i}\right)\;\left(Z_{j}\left(u\right)-m_{j}\right)\right]=\;=E\left[Z_{i}\left(u+h\right)\;Z_{j}\left(u\right)\right]-m_{i}\;m_{j},$$
  where u,u+h∈D×T,i,j=1,…,p.
  The function $C_{ij}\left(\cdot \right)$ is also called *direct covariogram*, if i=j, or *cross-covariogram*, if i≠j.

There exist several physical phenomena for which neither variance, nor the covariance exist, however it is possible to assume the existence of the variogram.

### 2.1. Separability for an *MSTRF*

The cross-covariance $C_{ij}$ for a second-order stationary *MSTRF* Z is *separable* if:

(3) $$C_{ij}\left(h\right)=\rho \left(h\right)\;a_{ij},\;h=\left(h_{s},h_{t}\right)\in D\times T,\;i,j=1,\ldots ,p,$$

where $a_{ij}$ are the elements of a (p×p) positive definite matrix and ρ(·) is a correlation function. In this case, it results in:

$$\frac{C_{ij}\left(h\right)}{C_{ij}\left(h^{\prime }\right)}=\frac{\rho (h)}{\rho (h^{\prime })},\;h,h^{\prime }\in D\times T,\;i,j=1,\ldots ,p,$$

hence the changes of the cross-covariances, with respect to the changes of the vector h, do not depend on the pair of the *STRF*$Z_{i},Z_{j}$.

The cross-covariance $C_{ij}$ for a second-order stationary *MSTRF* Z is *fully separable* if:

$$C_{ij}\left(h_{s},h_{t}\right)=\rho_{S}\left(h_{s}\right)\;\rho_{T}\left(h_{t}\right)a_{ij},\;\left(h_{s},h_{t}\right)\in D\times T,\;i,j=1,\ldots ,p,$$

where $a_{ij}$ are the elements of a (p×p) positive definite matrix, $\rho_{S}\left(\cdot \right)$ is a spatial correlation function and $\rho_{T}\left(\cdot \right)$ is a temporal correlation function. In the literature, many statistical tests for separability have been proposed and are based on parametric models [35,36,37], likelihood ratio tests and subsampling [38] or spectral methods [39,40].

### 2.2. Symmetry for an *MSTRF*

The cross-covariance $C_{ij}$ of a second-order stationary *MSTRF* Z, with *p* components, is *symmetric* if:

$$C_{ij}\left(h\right)=C_{ij}\left(-h\right),\;h\in D\times T,\;i,j=1,\ldots ,p,$$

or, equivalently, if:

$$C_{ij}\left(h\right)=C_{ji}\left(h\right),\;h\in D\times T,\;i,j=1,\ldots ,p.$$

The cross-covariance $C_{ij}$ of a second-order stationary *MSTRF* Z, with *p* components, is *fully symmetric* if:

$$C_{ij}\left(h_{s},h_{t}\right)=C_{ij}\left(h_{s},-h_{t}\right),\;\left(h_{s},h_{t}\right)\in D\times T,\;i,j=1,\ldots ,p,$$

or, equivalently,

$$C_{ij}\left(h_{s},h_{t}\right)=C_{ij}\left(-h_{s},h_{t}\right),\;\left(h_{s},h_{t}\right)\in D\times T,\;i,j=1,\ldots ,p.$$

Atmospheric, environmental and geophysical processes are often under the influence of prevailing air or water flows, resulting in a lack of full symmetry [26,41,42]. According to the relationships between separability, symmetry, stationarity and the *LCM* in the general class of cross-covariances of an *MSTRF* Z, it is worth recalling that if a cross-covariance is separable, then it is symmetric. However, in general, the converse is not true. Moreover, the hypothesis of full separability is a special case of full symmetry. Several tests to check the symmetry and separability of direct and cross-covariances can be found in the literature [24,40,43,44,45].

### 2.3. Direct and Cross-Covariance Estimators

Structural analysis requires covariance and variogram estimation, starting from the observed values of the variables under study. Hence, it is of basic importance to introduce the estimators of the direct and cross-covariances, respectively, defined afterwards.

Let $A_{i},i=1,\ldots ,p,$ be the sets of points of the domain $D\times T\subseteq R^{d+1}$, where the *p* variables have been observed: $A_{i}=\left\{u_{\alpha }={\left(s,t\right)}_{\alpha },\;\alpha =1,\ldots ,N_{i}\right\},\;i=1,\ldots ,p,$ then the estimators of the direct and cross-covariances are built as follows:

(4) $${\hat{C}}_{ij}\left(r_{s},r_{t}\right)=\frac{1}{|L_{ij}\left(r_{s},r_{t}\right)|}\sum_{L_{ij}\left(r_{s},r_{t}\right)}\left\{\left[Z_{i}\left(s+h_{s},t+h_{t}\right)-{\hat{m}}_{i}\right]\;\left[Z_{j}\left(s,t\right)-{\hat{m}}_{j}\right]\right\},$$

with i,j=1,…,p (i=j for the sample direct covariance and i≠j for the sample cross-covariance), where

$${\hat{m}}_{i}=\frac{1}{N_{i}}\sum_{\alpha =1}^{N_{i}}Z_{i}{\left(s,t\right)}_{\alpha },\;\;i=1,\ldots ,p,$$

while $|L_{ij}\left(r_{s},r_{t}\right)|$ is the cardinality of the following set:

$$L_{ij}\left(r_{s},r_{t}\right)=\left\{\left(s+h_{s},t+h_{t}\right)\in A_{i},\left(s,t\right)\in A_{j}:∥r_{s}-h_{s}∥<Tol\left(r_{s}\right)\;\mathrm{and}\;\left|r_{t}-h_{t}\right|<Tol\left(r_{t}\right)\right\},$$

where $r_{s}$ is the spatial separation vector (*lag*) with tolerance $Tol(r_{s})$ and $r_{t}$ is the temporal *lag* with tolerance $Tol(r_{t})$, then $Tol(r_{s})$ and $Tol(r_{t})$ are some specific regions of tolerance around $r_{s}$ and $r_{t}$, respectively.

## 3. Linear Coregionalization Model in Space–Time

ST-LCM is based on the hypothesis that each direct or cross-covariance function can be represented as a linear combination of some basic models and each direct or cross-covariance function must be built using the same basic models. Let Z be a second-order stationary *MSTRF* Z with *p* components, $Z_{i},\;i=1,\ldots ,p$. The matrix C for the second-order stationary Z is built as follows:

(5) $$C\left(h\right)=\sum_{l=1}^{L}\;B_{l}\;c_{l}\left(h\right).$$

where $h=(h_{s},h_{t})$, u=(s,t), $u+h=(s+h_{s},t+h_{t})$, $c_{l}$ are covariance functions, called *basic structures*, and  the matrices $B_{l}=\left[b_{ij}^{l}\right]$, called *coregionalization matrices*, must be positive definite, where the coefficients $b_{ij}^{l}$ satisfy the following property:

$$b_{ij}^{l}=b_{ji}^{l},\;i,j=1,\ldots ,p.$$

Thus, on the basis of this assumption $C_{ij}\left(h\right)=C_{ij}\left(-h\right)$ and $C_{ij}\left(h\right)=C_{ji}\left(h\right),$ with i,j=1,…,p,i≠j.

The basic structures $c_{l}\left(h\right)$$=c_{l}\left(h_{s},h_{t}\right)$ of the ST-LCM (5) can be modelled by using several space–time covariance models known in the literature, according to its empirical characteristics, which may necessitate the use of different classes of covariance models featuring various types of non-separability [33].

Fitting an ST-LCM to the data requires the identification of the space–time basic covariance functions and the corresponding positive definite coregionalization matrices. However, this is often a hard step to tackle. An approach based on the joint diagonalization of a set of covariance matrices computed for several spatio-temporal lags, allows us to determine the ST-LCM parameters in a very simple way.

### 3.1. Checking the Model Assumptions

In several environmental applications [10], the cross-covariance function is not symmetric, as for example, in time series in the presence of a delay effect, as well as in hydrology, for the cross-correlation between a variable and its derivative, such as water head and transmissivity [46]. Hence, this assumption should be tested before fitting an ST-LCM.

The appropriateness of the assumption of symmetry of an ST-LCM can be tested by using the methodology proposed by Li et al. [24] and discussed in De Iaco et al. [43]. This is based on the asymptotic joint normality of the sample space–time cross-covariances estimators. Given a set Λ of user-chosen spatio-temporal lags and the cardinality *c* of Λ, let $G_{n}=\left\{C_{ij}\left(h_{s},h_{t}\right):\left(h_{s},h_{t}\right)\in \Lambda ,i,j=1,\ldots ,p\right\}$ be a vector of $cp^{2}$ cross-covariances at spatio-temporal lags $k=(h_{s},h_{t})$ in Λ. Moreover, let ${\hat{C}}_{ij}\left(h_{s},h_{t}\right)$ be the estimator of $C_{ij}\left(h_{s},h_{t}\right)$ based on the sample data in the spatio-temporal domain $D\times T_{n}$, where *D* represents the spatial domain and $T_{n}=\left\{1,\ldots ,n\right\}$ the temporal one, and  define $\left\{{\hat{C}}_{ij}\left(h_{s},h_{t}\right):\left(h_{s},h_{t}\right)\in \Lambda ,i,j=1,\ldots ,p\right\}$. Under the assumptions given in Li et al. [24], $|T_{n}{|}^{1/2}\left({\hat{G}}_{n}-G\right)\overset{d}{\to }N_{cp^{2}}\left(0,\Sigma \right)$, where $|T_{n}|\Sigma$ converges to $Cov({\hat{G}}_{n},{\hat{G}}_{n})$. The tests for symmetry properties can then be based on the following statistics

(6) $$TS=|T_{n}|{\left(A{\hat{G}}_{n}\right)}^{T}{\left(A\Sigma A^{T}\right)}^{-1}\left(A{\hat{G}}_{n}\right)\overset{d}{\to }\chi_{a}^{2},$$

where *a* is the row rank of the matrix A, which is such that AG=0 under the null hypothesis.

Moreover, the choice of modeling the *MSTRF* Z by an ST-LCM is based on the prior assumption that the multivariate correlation structure of the variables under study is characterized by L(L≥2) scales of spatio-temporal variability. On the other hand, if the multivariate correlation of a set of variables does not present different scales of variability (L=1), then the cross-covariance functions are separable. (3) Hence, as in the spatial context [10], a space–time intrinsic coregionalization model can be considered. Obviously, this last model is just a particular case (L=1) of the ST-LCM defined in (5) and it is much more restrictive than the linear model [34] since it requires that all the variables have the same correlation function, with possible changes in the sill values. Note that, if a cross-covariance is separable, then it is symmetric.

Once the basic components $c_{l},l=1,\ldots ,L$, are estimated, it is necessary to proceed with their modeling. The choice of a reasonable class of models to be fitted to each empirical component ${\hat{c}}_{l}$ can be supported by analyzing the characteristics of the empirical basic covariance surfaces [47], such as the type of non-separability through the computation of the sample non-separability ratios [14] or by applying a statistical test [48,49].

**Remark 1.**  *In the ST-LCM, each component is represented as a linear combination of latent, uncorrelated univariate spatio-temporal processes. However, the smoothness of any component defaults to that of the roughest latent process, and thus the standard approach does not admit individually distinct smoothness properties, unless structural zeros are imposed on the latent process coefficients [9].*

### 3.2. Some Computational Aspects on the ST-LCM Fitting

The model in (5) can be fitted by following a simplified procedure. First, the  empirical basic covariance functions are detected through the use of the joint diagonalization of the sample (p×p) covariance matrices $\hat{C}{\left(h_{s},h_{t}\right)}_{k}=\left[{\hat{C}}_{ij}{\left(h_{s},h_{t}\right)}_{k}\right]$ evaluated at different space–time lags ${\left(h_{s},h_{t}\right)}_{k}=\left(h_{s_{k}},h_{t_{k}}\right)$, with k=1,…,K. In particular, after determining the (p×p) orthogonal matrix Ψ, such that

(7) $$\Psi \;\hat{C}{\left(h_{s},h_{t}\right)}_{k}\;\Psi^{T}=\Delta {\left(h_{s},h_{t}\right)}_{k},\;k=1,\ldots ,K,$$

where $\Delta_{k}$ are the diagonal (p×p) matrices, the sample basic uncorrelated components ${\hat{c}}_{l}$ (estimates of $c_{l},l=1,\ldots ,p$) are obtained by extracting all the diagonal entries across the *K* matrices $\Delta_{k}$ [50,51]. Joint diagonalization with respect to the lags implies that the matrix Ψ does not depend on the lags. For the purpose of joint diagonalization many algorithms exist, see Illner et al. [52] for an overview. In this study, the algorithm based on Jacobi rotations [53] and included in the R package JADE [54] is recalled. Note that only the L≤p basic components ${\hat{c}}_{l},l=1,\ldots ,L,$ are included in the ST-LCM; the selected components are the ones that exhibit distinct spatio-temporal scales of variability (corresponding to the lag where the surface decays).

These selected basic components are then modelled by adopting appropriate classes of models (according to the empirical characteristics of each basic component). At the end, the  coregionalization matrices are computed and their admissibility is checked. A reasonable class of models to be fitted to each component can be assessed, according to some characteristics, such as full symmetry ($c_{l}\left(h_{s},h_{t}\right)=c_{l}\left(h_{s},-h_{t}\right)=c_{l}\left(-h_{s},h_{t}\right)$) and full separability ($c_{l}\left(h_{s},h_{t}\right)=c_{l}\left(h_{s}\right)c_{l}\left(h_{t}\right)/c_{l}\left(0,0\right)$), which the sample covariance surfaces might satisfy. In the case of non-separability, the type of non-separability can be studied through computation of the sample non-separability ratios, as in [14], which measure the discrepancy between the sample covariance function (supposed non-separable) and the one corresponding to the separable case (i.e., the product of the spatial and temporal marginals). Some statistical tests given in [48,49] can be easily applied toward this aim, without assuming any specific distribution for the data.

At the end, the elements $b_{ij}^{l}$ of $B_{l},l=1,\ldots ,L,$ of the model in (5) are estimated. They correspond to the ratio between the contributions of ${\hat{C}}_{ij}$ at the *l*-th scale of variability, by $\left[c_{l}\left(0,0\right)\right]$, i.e.,

(8) $$b_{ij}^{l}=\frac{\left[{\hat{C}}_{ij}{\left(h_{s},h_{t}\right)}_{l-1}\right]-\left[{\hat{C}}_{ij}{\left(h_{s},h_{t}\right)}_{l}\right]}{\left[c_{l}\left(0,0\right)\right]},\;l=1,\ldots ,L,$$

where ${\hat{C}}_{ij}{\left(h_{s},h_{t}\right)}_{0}={\hat{C}}_{ij}\left(0,0\right),$ with i,j=1,…,p,i≤j.

The positive definiteness condition of the matrices $B_{l},l=1,\ldots ,L,$ is verified by checking that their eigenvalues are non negative. In particular, after performing the spectral decomposition of these matrices,

$$B_{l}=V_{l}\Lambda_{l}V_{l}^{T},\;l=1,\ldots ,L,$$

where $V_{l}$ are the eigenvector matrices and $\Lambda_{l}$ the diagonal matrices of the eigenvalues, if there are some negative eigenvalues, they are set equal to zero. In this case, the transformed coregionalization matrix $B_{l}^{\prime }$ is derived through the following expression

(9) $$B_{l}^{\prime }=V_{l}\Lambda_{l}^{\prime }V_{l}^{T}\;l=1,\ldots ,L,$$

where the diagonal matrix of the eigenvalues $\Lambda_{l}^{\prime }$ is modified with respect to the original $\Lambda_{l}$ since zeros are in place of the negative eigenvalues.

Further details on computational aspects can be found in [55].

## 4. Prediction and Risk Assessment in Space–Time

For prediction purposes, various cokriging algorithms can be found in the literature [56,57]. As a natural extension of spatial ordinary cokriging to the spatio-temporal context, the linear space–time predictor can be written as

(10) $${\hat{Z}}_{i}\left(u\right)=\sum_{j=1}^{p}\sum_{\alpha =1}^{N_{j}}\;w_{\alpha }^{ij}\left(u\right)Z_{j}\left(u_{\alpha }\right),\;\;i=1,\cdots ,p$$

where u=(s,t)∈D×T is a point in the space–time domain, $u_{\alpha }={\left(s,t\right)}_{\alpha }\in D\times T,$ $\alpha =1,\ldots ,N_{i},$ are the data points in the same domain and $w_{\alpha }^{ij}\left(u\right)$ are the weights assigned to the value of the *j*th variable, j=1,…,p, at the αth data point, to predict the *i*th variable, i=1,…,p, at the point u∈D×T.

The predicted space–time random vector $\hat{Z}\left(u\right)$ at u∈D×T, is such that each component ${\hat{Z}}_{i}\left(u\right),\;i=1,\ldots ,p,$ is obtained by using all information at the data points $u_{\alpha }={\left(s,t\right)}_{\alpha }\;\in D\times T,\;\alpha =1,\ldots ,N_{i}$.

The weights $w_{\alpha }^{ij}\left(u\right),\;\alpha =1,\ldots ,N_{i},$ are determined by ensuring the unbiased condition for the predictor $\hat{Z_{i}}\left(u\right)$ and the efficiency condition, obtained by minimizing the error variance [34].

Similarly, for environmental risk assessment, the  formalism of multivariate spatio-temporal indicator random function (MSTIRF) and corresponding predictor, have to be introduced. Let

$$I\left(u,z\right)={\left[I_{1}\left(u,z_{1}\right),\ldots ,I_{p}\left(u,z_{p}\right)\right]}^{T},$$

be a vector of *p* spatio-temporal indicator random functions (*STIRF*) defined on the domain $D\times T\subseteq R^{d+1}$, with (d≤3), as follows

$$I_{i}\left(u,z_{i}\right)=\left\{\begin{array}{cc} 1 & \;\mathrm{if}\;Z_{i}\mathrm{is}\;\mathrm{not}\;\mathrm{greater}\;\left(\mathrm{or}\;\mathrm{not}\;\mathrm{less}\right)\;\mathrm{than}\;\mathrm{the}\;\mathrm{threshold}\;z_{i},\\ 0 & \;\mathrm{otherwise} \end{array}\right.$$

where $z={\left[z_{1},\ldots ,z_{p}\right]}^{T}$. Then

$$\{I\left(u,z\right),\;u=\left(s,t\right)\in D\times T\subseteq R^{d+1}\},$$

represents an *MSTIRF*. In other words, for each coregionalized variable $Z_{i},$ with i=1,…,p, an *STIRF* $I_{i}$ can be appropriately defined. Then the linear space–time predictor (10) can be easily written in terms of the indicator random variables $I_{i},\;i=1,\ldots ,p$. If the spatio-temporal correlation structure of an MSTIRF is modelled by using the ST-LCM, the cokriging can be used to produce risk assessment maps, for one or all the variables under study. If p=1, the dependence of the indicator variable is characterized by the corresponding indicator covariance of *I*: $C_{{}_{ST}}\left(h\right)$, which depends solely on the lag vector $h=(h_{s},h_{t})$, for any pair of points (s,t) and $\left(s+h_{s},t+h_{t}\right)$ where $\left(s,s+h_{s}\right)\in D^{2}$ and $\left(t,t+h_{t}\right)\in T^{2}$. The fitted model for $C_{{}_{ST}}$ must satisfy an admissibility condition in order to be valid and ordinary kriging can be used to generate the environmental risk assessment maps.

The GSLib routine “COK2ST” [29] can be used to produce multivariate predictions in space–time, for one or all the variables under study, using the ST-LCM (5).

## 5. Space–Time Multivariate Analysis for Radon Flux Measurements

In this section, after a brief description of the geographical area under study, in terms of geological characteristics and meteorological conditions, the spatio-temporal multivariate data set related to the variables radon flux, average temperature, minimum humidity and evapotranspiration is presented. Finally, a description of the spatial and temporal profile of the variables is proposed.

### 5.1. Study Area

**Figure 1:**
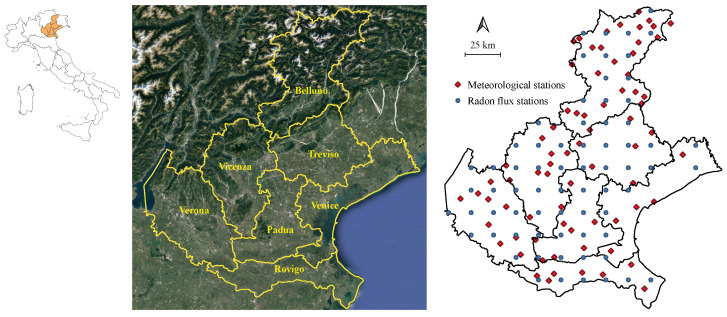
(**Left panel**): map of Italian regions (study area in orange). (**Middle panel**): Veneto Provinces. (**Right panel**): location map of meteorological and radon sample points over the study area.

The Veneto Region is located in the north-eastern part of Italy and occupies an area of about 18,400 km${}^{2}$. It is divided into seven provinces (Belluno, Padua, Rovigo, Treviso, Venice, Verona and Vicenza) as shown in Figure 1.

Veneto’s geomorphology setting is very composite: 57% of its surface is covered by plain, 29% is mountainous (i.e., the Carnic Alps, eastern Dolomites and Venetian Prealps, in the northern zone), and the remaining 14% is constituted by hills. The Veneto Region is crossed by several of the most important rivers in Italy (i.e., the Adige, Brenta, Piave, Po and Tagliamento) and it possesses the eastern shore of the Lake Garda. The permeability of the lithologies varies from low and moderately low in the plain area, characterized by sandy and silty-clay deposits, to moderately high and high in the south-west and north of the region, due to the presence of limestone, sandstone and calcarenite soils. Finally, the large number of faults indicates intense tectonic activity in the region.

With regards to the climate, in Veneto two climatic zones can be detected: the Alpine region, which is characterized by mild summers and cold temperatures in winter with frequent snowfalls, on the other hand hilly and plain areas have a continental climate, with hot summers and very cold winters.

A wide variety of geological features and meteorological conditions which characterize the Veneto Region, strongly contribute to the exhalation of the radon from the soil, hence studies of radon flux over this region might be very interesting. To the best of our knowledge, up to now only few studies have referred to indoor radon concentrations in Veneto [58,59,60], based on the monitoring campaigns conducted starting from 1989, with the aim of assessing human health risks. However, these monitoring campaigns were discontinuous over time, and mainly regarded the risk areas identified in 1989 and only a few buildings. Although atmospheric radon flux does not represent a health risk, for smaller magnitudes than those of indoor radon, an accurate evaluation of its exhalation rate could be relevant for epidemiological studies and to determine emission strategies of greenhouse gases. Furthermore, outdoor radon and radon flux represent an important factor for the correction of indoor values [1,2].

In the present paper, a geostatistical analysis of the spatio-temporal behavior of radon flux jointly with three meteorological variables, which affect the gas dispersion [61,62], has been proposed. As pointed out in [63,64], many meteorological variables can affect radon exhalation from soil; for this reason, the average air temperature, minimum humidity and evapotranspiration have been considered. In the following spatio-temporal multivariate analysis of radon flux, the prediction maps of environmental radioactivity over the Veneto Region might contribute to an accurate assessment of the indoor radon levels, as well as to the identification of radon priority areas [1,2] and might be used by local or regional authorities for land-use planning and urban development. The latter aspect is very important especially considering that, ranking fourth in Italy, Veneto has a population of over 4.8 million inhabitants, with a yearly increase in population growth rate of 0.6‰ (higher with respect to the Italian population growth rate equal to 0.4‰).

### 5.2. Spatio-Temporal Multivariate Data Set

The multivariate spatio-temporal data set involves monthly values of radon flux (Rn flux, in KBqm${}^{-2}$ s${}^{-1}$), which is a measure of radon exhaled per surface unit and per time unit, as well as monthly averages of evapotranspiration ($ET_{0}$, in mm), minimum humidity ($H_{m}$, in %) and mean temperature ($T_{M}$, in °C) observed over Veneto Region (Italy), from January 2006 to April 2022 (i.e., 196 temporal observations). As can be seen in Figure 1, the meteorological variables ($T_{M}$, $H_{m}$ and $ET_{0}$) were measured at 72 survey stations belonging to the agency for environmental protection and downloaded from https://www.arpa.veneto.it/ ((accessed on 25 June 2023). On the other hand, the monthly Rn flux data referred to 69 spatial points regularly distributed over a grid with 15 km × 15 km cell size. It is worth noting that the Rn exhalation rate is freely downloadable from https://meta.icos-cp.eu/objects/5-Z-zRaqFgddALv0ohLonzWD (accessed on 25 June 2023) for the whole European land surface. Rn flux data have been computed by using the measurements of soil properties, uranium content as well as model-derived soil moisture and water-table depth, as described in [65].

### 5.3. Exploratory Data Analysis

**Figure 2:**
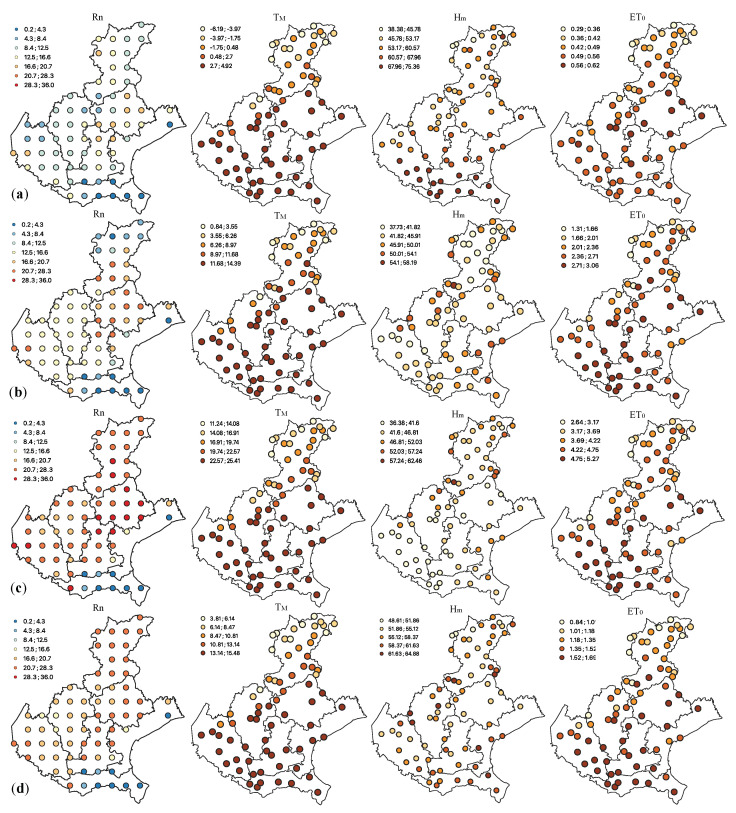
Colour maps of Rn flux (in KBqm${}^{-2}$ s${}^{-1}$), $T_{M}$ (in °C), $H_{m}$ (in %) and $ET_{0}$ (in mm) monthly averages calculated for (**a**) January, (**b**) April, (**c**) July and (**d**) October.

In Figure 2, the spatial representation of Rn flux and of the three meteorological variables has been provided, by computing the monthly averages in January, April, July and October for the analyzed 17-year span. The represented values for the fixed four months can be considered representative of the behavior of the variables of interest over the domain during the four seasons of a year.

The Rn flux areas with high gas exhalation rates, for the fixed four months, are located in the Province of Belluno and in the west of Verona. Moreover, some isolated areas with high radon levels have also been identified in the Province of Treviso, bordering Friuli Venezia Giulia, and in the Province of Padua (i.e., the Euganean Hills area, which is characterized by a complex geological context). Note that, as pointed out in [58,60] the same areas are also characterized by high indoor radon concentrations. The highest average values have been observed in July (mean and median equal to 22.10 and 23.62 KBqm${}^{-2}$ s${}^{-1}$, respectively, and coefficient of variation equal to 53.86%). On the other hand, the lowest values (from 0.18 to 20.56 KBqm${}^{-2}$ s${}^{-1}$) have been observed in January (mean and median values equal to 11.53 and 12.21 KBqm${}^{-2}$ s${}^{-1}$, respectively, and coefficient of variation equal to 40.01%). It is worth noting that the exhalation rates increase during the summer period, when $T_{M}$ and $ET_{0}$ increase and $H_{m}$ decreases [2].

Regarding the meteorological variables ($T_{M}$, $H_{m}$ and $ET_{0}$), Figure 2 shows their spatial behavior that changes significantly from the plain to the mountains areas.

The differences in terms of $T_{M}$ between the high mountains and the plain and coastal areas are quite large. The $T_{M}$ in January varies from 2.7 to 4.92 °C along the plain and coast, while in the mountainous zones it can be very low during winters (from −6.19 to −3.97 °C); wider differences are also evident in July.

The Veneto Region climate is characterized by high humidity levels, indeed the percentage of $H_{m}$ is very high in the 73 monitoring stations, especially in January and October, with a minimum percentage between 38.38 and 48.61, respectively, and maximum percentage ranging between 64.88 (in October) and 75.36 (in January).

Looking at the colour maps of the $ET_{0}$ (Figure 2), which represents a combination of evaporation and transpiration processes of water from the soil to the air, low levels have been observed in the northern and north-eastern part of the Veneto Region and high in the plain. In January, the values vary from 0.29 mm on the mountainous interior zones to 0.62 mm in the Treviso Province. On the other hand, in July the $ET_{0}$ levels are high all over the domain with peaks in the central part (plain areas). Moreover, the well-known positive correlation between air temperature and evapotranspiration, as well as the negative correlation between air temperature (or evapotranspiration) and humidity were confirmed via the spatial profile analyses. In addition, in the central part of the study area the geological characteristics of the soil as well as meteorological and climatic condition affect Rn exhalations from the soil to the atmosphere.

**Figure 3:**
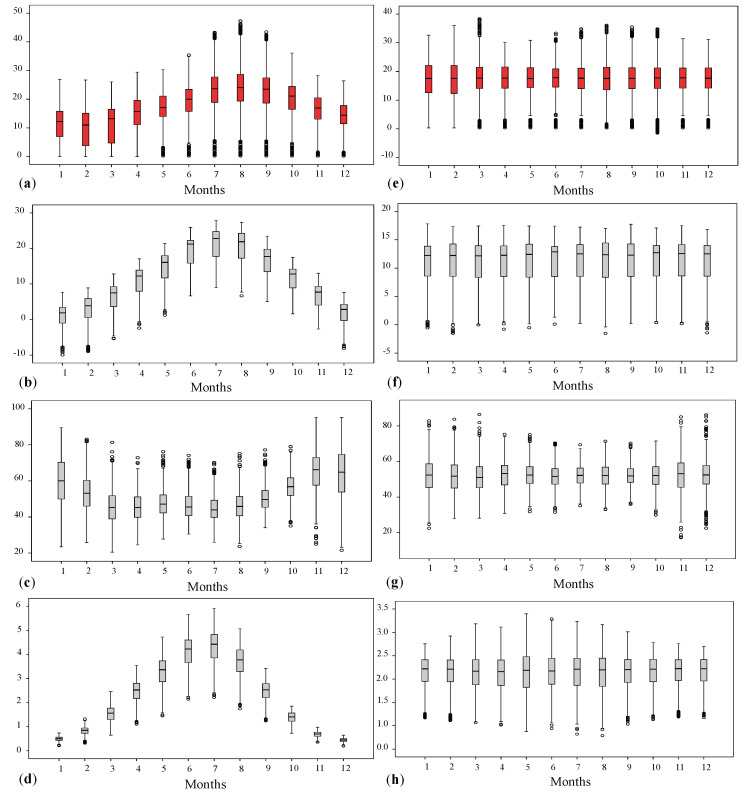
Box and whisker plots showing (**a**) Rn flux (in KBqm${}^{-2}$ s${}^{-1}$), (**b**) $T_{M}$ (in °C), (**c**) $H_{m}$ (in %) and (**d**) $ET_{0}$ (in mm) and their corresponding residual values (**e**–**h**), grouped by month. The symbol ∘ indicates values which lie more than 1.5 times the interquartile range from the first and third quartile.

The temporal profiles of the analyzed variables have been evaluated through box plots of the monthly values at the sample points (Figure 3a–d). All the four variables exhibit a seasonal behavior: Rn flux, $T_{M}$ and $ET_{0}$ are characterized by increasing values during the spring and summer periods, on the other hand the $H_{m}$ denotes an opposite behavior, with increasing values during autumn/winter and decreasing values during spring/summer. Moreover, as previously pointed out for the spatial profile analyses, it is evident that low (high) values of Rn flux are associated to low (high) values of the $T_{M}$ and $ET_{0}$ and to high (low) $H_{m}$ [2].

Since the time series of the analyzed variables exhibit a seasonal component, the twelve-month averages have been computed for each station and the periodic component of each variable has been removed from the monthly values by subtracting the average seasonality. In Figure 3e–h the deseasonalized values of the four variables, grouped by months, are shown.

Then, as described in Section 6, the spatio-temporal direct and cross-covariance functions of the residuals of Rn flux, $T_{M}$, $H_{m}$ and $ET_{0}$ were analyzed and an appropriate ST-LCM was selected.

Space–time modeling and prediction techniques were applied in order to forecast the Rn flux values over the area of interest for the period May–December 2022 (i.e., the months after the last available time point in the analyzed data set). In particular, the following aspects were considered:

(1) estimating and modeling space–time correlation among the residuals’ variables; in the ST-LCM fitting stage the procedure proposed in [30] and based on the joint diagonalization of several sample covariance matrices was performed and the most apt covariance model [66] was fitted for each basic component;
(2) predicting Rn flux during the period May–December 2022 by using spatio-temporal cokriging based on the estimated ST-LCM;
(3) producing risk maps showing the probability that Rn flux in a summer month exceeds the value of some chosen statistics, by using indicator kriging [67].

## 6. Modeling the ST-LCM for the Study Variables

Modeling the spatio-temporal correlation among the variables under study by using the ST-LCM, requires first checking the adequacy of such a model. In particular, the symmetry assumption was checked by using the methodology proposed by [24] and mentioned in Section 3.1. Therefore, after selecting three pairs of spatial points and the temporal lag equal to 1 month, which is the lag corresponding to the largest empirical cross-correlations for all variables combinations, the test statistic TS (6) was equal to 0.34 with a corresponding *p*-value equal to 0.99. On the basis of this result, it is reasonable to consider the ST-LCM a suitable model for the data set under study.

**Figure 4:**
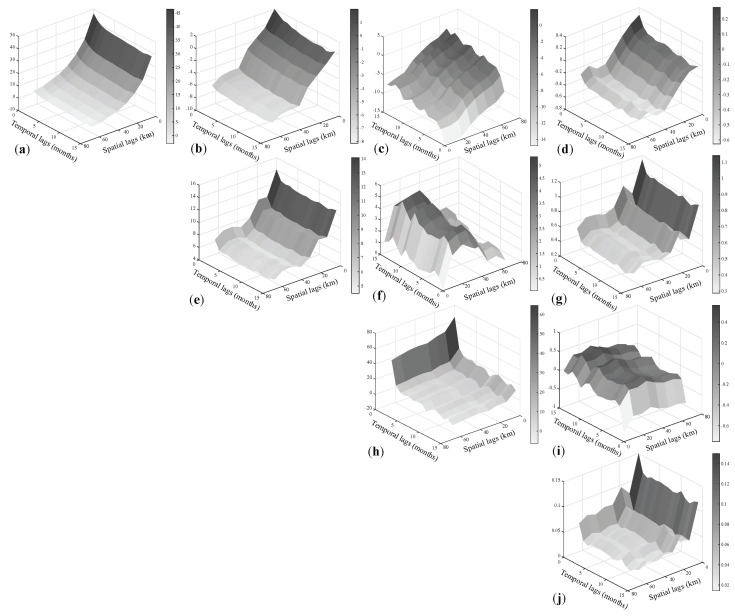
Sample space–time direct covariance surfaces for the residuals of (**a**) Rn flux, (**e**) $T_{M}$, (**h**) $H_{m}$ and (**j**) $ET_{0}$ together with the cross-covariance surfaces of (**b**) Rn flux vs. $T_{M}$, (**c**) Rn flux vs. $H_{m}$, (**d**) Rn flux vs. $ET_{0}$, (**f**) $T_{M}$ vs. $H_{m}$, (**g**) $T_{M}$ vs. $ET_{0}$, (**h**) $H_{m}$ and (**i**) $H_{m}$ vs. $ET_{0}$, computed on the basis of the estimator in (4).

Through the fitting procedure developed by [33] and starting from the estimation of the sample covariance matrices computed for a set of spatio-temporal lags and by using the outputs from the joint diagonalization of these covariance matrices, the uncorrelated basic components underlying the investigated phenomenon were identified. In the present case study, according to the geometry of the spatio-temporal domain, 8 spatial lags and 15 temporal lags for a total of 120 spatio-temporal lags, were fixed to estimate the 4 direct covariance functions and 6 cross-covariance functions, whose surfaces are shown in Figure 4. Note that all the empirical direct covariance surfaces show a strong linear relationship for short and medium lags in space–time, while decaying otherwise; from the empirical cross-covariance surfaces, it is evident the presence of a negative linear relationship between Rn flux and $H_{m}$ for short and medium lags as well as between $H_{m}$ and $ET_{0}$ for short lags, while it is positive in the other cases, as can be reasonably justified from their natural characteristics.

Successively, the 120 symmetric matrices of the sample direct and cross-covariances (matrices with dimension 4×4) have been jointly diagonalized (as previously mentioned, the diagonalization was carried out using the R package JADE) and 120 diagonal matrices plus the following orthogonal matrix Ψ were found out

$$\Psi =\left[\begin{array}{cccc} 0.92644863 & -0.34811734 & 0.14315315 & 0.003798338\\ 0.36544978 & 0.91846172 & -0.12952119 & -0.078094773\\ -0.08630179 & 0.17383656 & 0.98088864 & 0.013795947\\ 0.02629437 & 0.07087482 & -0.02426757 & 0.996843243 \end{array}\right].$$

**Figure 5:**
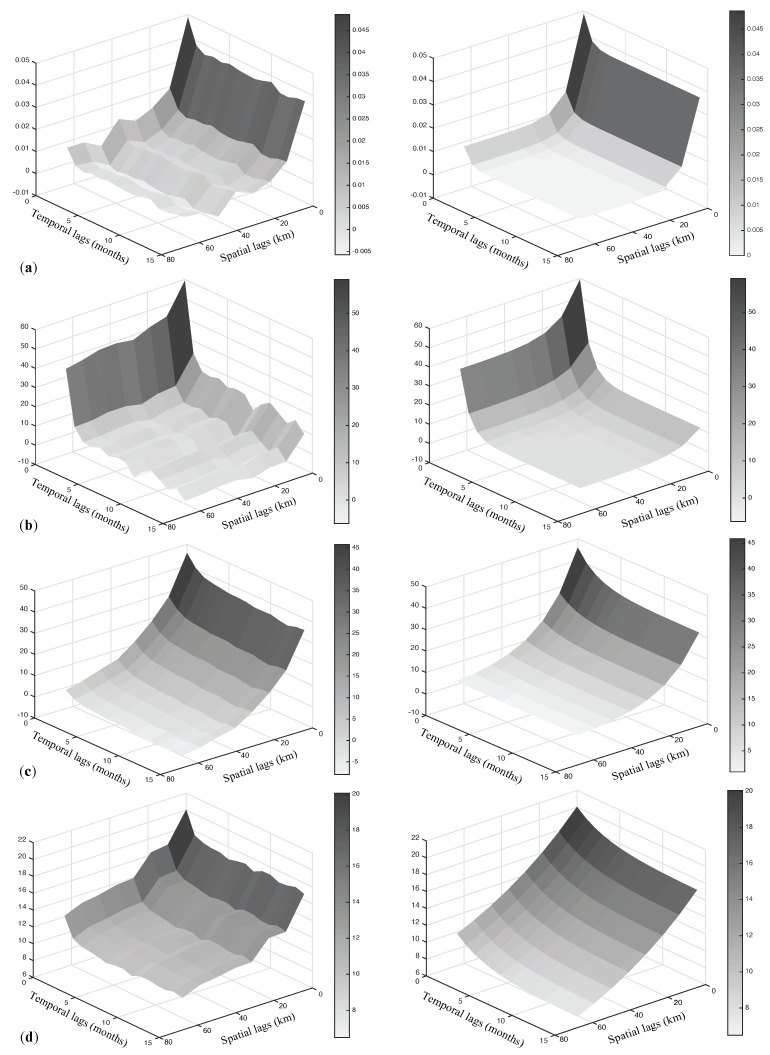
Sample spatio-temporal covariance surfaces (on the left) and fitted models (on the right), for the basic components at (**a**) very small, (**b**) small, (**c**) medium and (**d**) large scale of spatio-temporal variability.

From the obtained 120 diagonal matrices, the sample basic uncorrelated components ${\hat{c}}_{l}$, which correspond to the estimates of $c_{l}\;\left(l=1,\ldots ,4\right)$, were determined by extracting all the diagonal entries across the 120 matrices. Through a graphical check of the ${\hat{c}}_{l}$ surfaces (the 3D plots shown in Figure 5), the following distinct scales of spatio-temporal variability, i.e., the distance in space and time at which the surface decays, have been detected:

- 20 km in space and 2 months in time (very small scale),
- 30 km in space and 3 months in time (small scale),
- 55 km in space and 7 months in time (medium scale),
- 120 km in space and 12 months in time (large scale).

The different behaviors in space and time of the basic components have suggested to retain all of them and proceed to construct the ST-LCM with four uncorrelated components, as follows:

(11) $$C\left(h\right)=B_{1}\;c_{1}\left(h\right)+B_{2}\;c_{2}\left(h\right)+B_{3}\;c_{3}\left(h\right)+B_{4}\;c_{4}\left(h\right),$$

where the coregionalization matrices $B_{l},l=1,\ldots ,4,$ have to be computed as indicated in (8) and the basic components $c_{l}\left(h\right),l=1,\ldots ,4,$ have to be modelled after identifying the most apt class of covariance models with respect to some features (full symmetry, non-separability and type of non-separability) of the sample basic covariance surfaces. For this last aim, the statistical tests for symmetry and separability, based on the asymptotic joint normality of the sample space–time covariance estimators [68], were carried out according to the procedure in [48,49], for each basic component. In the same papers, the details on the test statistics, denoted by $TS_{1}$ and $TS_{2}$, and the corresponding probability distribution were given.

**Figure 6:**
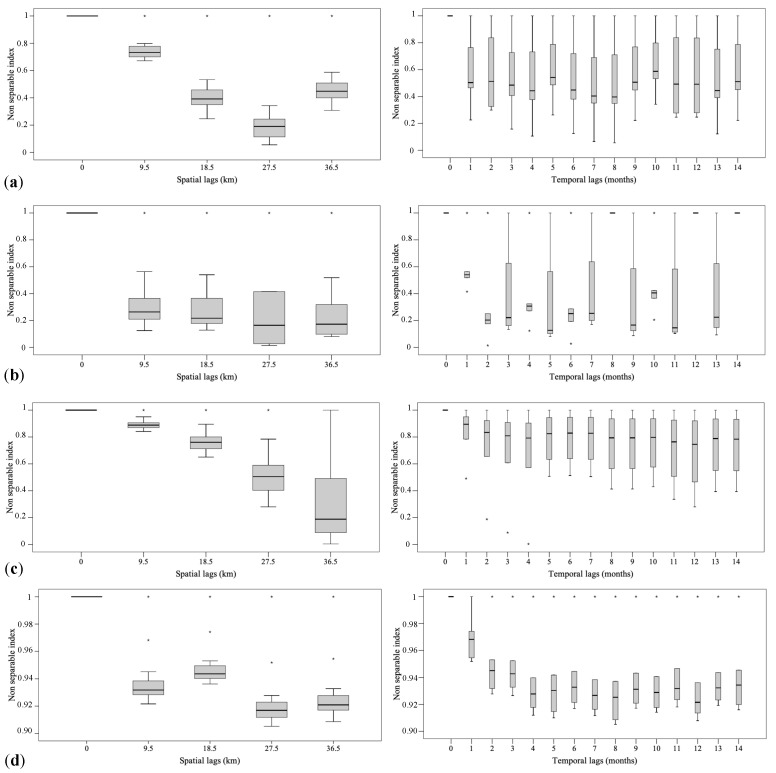
Box and whisker plots of sample non-separability ratios classified by spatial (on the left) and temporal (on the right) lags, computed for the basic components at (**a**) very small (**b**) small, (**c**) medium and (**d**) large scale of spatio-temporal variability. The symbol * indicates values which lie more than 1.5 times the interquartile range from the first and third quartile.

From these tests’ results, it was possible to conclude that at 5% significance level, the null hypothesis of full symmetry cannot be rejected for all basic components and the null hypothesis of separability can be rejected for all basic components. With regard to the type of non-separability, the non-separability ratios, defined in [14], have been calculated for the spatial and temporal lags for which correlation in space and time is stronger, and the corresponding values have been summarized through the box and whisker plots shown in Figure 6. These graphs are very useful tools to establish the type of non-separability: in this case study the non-separability ratios grouped by spatial and temporal lags have been always smaller than one, hence a uniform negative non-separability assumption is reasonable for the four basic components.

From the obtained results, the class of fully symmetric and uniform negative non-separable covariance models has been the most appropriate class of models for each $c_{l}$. In particular, the product–sum covariance function has been adopted, i.e.,

(12) $$c_{l}\left(h_{s},h_{t}\right)=k_{1_{l}}C_{s_{l}}\left(h_{s}\right)C_{t_{l}}\left(h_{t}\right)+k_{2_{l}}C_{s_{l}}\left(h_{s}\right)+k_{3_{l}}C_{t_{l}}\left(h_{t}\right),\;l=1,\ldots ,4,$$

with $C_{s_{l}}=Exp(||h_{s}||;a_{l})$ the spatial exponential covariance model in $R^{d}$ with practical range $a_{l}$, $C_{t_{l}}=Exp(|h_{t}\left|;b_{l}\right)$ the temporal exponential covariance model in R, with practical range $b_{l}$, and parameters $k_{1_{l}},k_{2_{l}}$ and $k_{3_{l}},l=1,\ldots ,4,$ as reported in Table 1. This kind of covariance model is widely used not only in environmental sciences but also in other scientific fields, such as in Demography [69]. These estimates ensure the strict positive definiteness of the basic models [70].

Finally, the matrices $B_{l},l=1,\ldots ,4,$ whose entries were computed by the expression in (8), include the following:

$$B_{1}=\left[\begin{array}{cccc} 663.8366 & 86.7824 & -121.4861 & 8.5663\\ 86.7824 & 109.8702 & -89.6217 & 9.3176\\ -121.4861 & -89.6217 & 1214.1283 & -21.6994\\ 8.5663 & 9.3176 & -21.6994 & 1.8972 \end{array}\right],\;B_{2}=\left[\begin{array}{cccc} 0.1100 & 0.0236 & -0.0443 & 0.0029\\ 0.0236 & 0.0380 & 0.0057 & 0.0037\\ -0.0443 & 0.0057 & 0.0601 & -0.0005\\ 0.0029 & 0.0037 & -0.0005 & 0.0004 \end{array}\right],\;B_{3}=\left[\begin{array}{cccc} 0.2043 & 0.0537 & -0.0640 & 0.0012\\ 0.0537 & 0.0485 & 0.0156 & 0.0029\\ -0.0640 & 0.0156 & 0.0520 & 0.0020\\ 0.0012 & 0.0029 & 0.0020 & 0.0040 \end{array}\right],\;B_{4}=\left[\begin{array}{cccc} 0.4020 & -0.2880 & -0.1704 & -0.0200\\ -0.2880 & 0.5900 & 0.2110 & 0.0100\\ -0.1704 & 0.2110 & 0.4000 & 0.0116\\ -0.0200 & 0.0100 & 0.0116 & 0.0011 \end{array}\right].\;$$

Note that the above coregionalization matrices are all positive definite, i.e., the corresponding eigenvalues are non-negative, thus satisfying the admissibility condition for the fitted ST-LCM.

In the next stage of the analysis, the adequacy of model (11) will be evaluated, and then the same model will be used to produce spatio-temporal predictions of the Rn flux.

The detection of the uncorrelated components, the identification of an apt covariance model for each component, as well as the computation of coregionalization matrices can be realized in the R environment, recalling properly defined functions, which are available upon request from the corresponding author.

### Adequacy of the Fitted Model

The suitability of the fitted ST-LCM was assessed using a three-fold procedure, i.e.,

(a) a comparative analysis with respect to the intrinsic coregionalization model, defined from the ST-LCM by neglecting the presence of different scales of spatio-temporal variability,
(b) the leave-one-out cross-validation technique and the computation of the linear correlation coefficient among the available data for the Rn flux and their estimates,
(c) the jackknife prediction of Rn flux for the last four available months (January, February, March and April 2022), whose data have not been used in the previous structural analysis, and the comparison of the predicted values with respect to the true ones.

With regard to point (a), an intrinsic coregionalization model, namely an ST-LCM with only one basic component, has been chosen as alternative contender model. In particular, it has been assumed that the study data set did not present different scales of variability in space and time, and only the basic component with the largest scale of spatio-temporal variability (20 km in space and 12 months in time) was common to the investigated variables. Hence, the following model has been considered:

(13) $$C^{\prime }\left(h\right)=B\;c\left(h\right),$$

where the unique basic component has been modelled by the product–sum covariance function with a spatial exponential covariance model whose practical range is equal to 120 km, a temporal exponential covariance model with practical range equal to 12 months, and the parameter $k_{1},k_{2}$ and $k_{3}$ equal, respectively, to 0.010, 17.0644 and 2.9732. The following coregionalization matrix

$$B=\left[\begin{array}{cccc} 2.8031 & 0.1150 & -0.4013 & 0.0122\\ 0.1150 & 1.0793 & 0.0464 & 0.0501\\ -0.4013 & 0.0464 & 3.2720 & -0.0378\\ 0.0122 & 0.0501 & -0.0378 & 0.0160 \end{array}\right],$$

has been estimated to complete the contender model in (13).

The fitting goodness of the two ST-LCM has been measured on the basis of the errors between the sample covariance values and the theoretical ones, for the first 5 spatial lags and 8 temporal lags, which represent the lags where the correlation is reasonably stronger. In particular, the Root Average Error (RAE), defined by [71] as the square root of the ratio between the sum of the squared errors and the sum of the squared sample values, as well as the Relative Mean Absolute Error (RMAE), computed as the ratio between the sum of the absolute errors and the sum of the absolute sample values, were produced and the results are reported in Table 2.

The error metrics’ values are almost always greater in the case of the model with a unique basic component; these results have highlighted the adequacy of the ST-LCM with four basic components, while model (13) has determined the worst fitting for the study variables. In other words, the ST-LCM with four basic product–sum models has better described the multivariate spatio–temporal correlation which characterized the investigated phenomenon.

The second check of the suitability of the fitted model in (11), performed through the leave-one-out cross-validation technique, required the computation of the cokriging estimations for the Rn flux residuals. Spatio-temporal cokriging was implemented by alternatively using the ST-LCMs (11) and (13), and then calculating the correlation coefficients between the Rn flux estimates and the recorded values. Thus, in the case of model (11) the correlation coefficient was equal to 0.972 (significant at 1% level). On the other hand, for the model (13) a correlation coefficient equal to 0.773 was found. Evidently, the adequacy of the ST-LCM with four basic components fitted by the product–sum covariance models was also confirmed by the cross-validation results.

Finally, the last procedure to assess the adequacy of the ST-LCM (11) was developed in order to make jackknife predictions of the Rn flux levels in the last four months (January, February, March and April 2022), where the available data have been used as a test set. The predictions have been computed through, alternatively

- the spatio-temporal cokriging based on the ST-LCM in (11),
- the spatio-temporal cokriging based on the intrinsic coregionalization model in (13),
- the spatio-temporal kriging based on the product–sum covariance model of the Rn flux residuals, already included in the ST-LCM (11).

Rn flux residuals were first obtained through the above interpolation techniques and, successively, the Rn flux seasonal components were added to the forecasts.

On one hand, the final results were then compared on the basis of the correlation coefficient calculated among true values and predicted ones, and on the other hand, by the error measures RAE and RMAE since they furnish a measure of the relative average discrepancy between the true values of Rn flux and its predictions. In Table 3, all these statistics have been summarized, from which it is evident the better performance of the cokriging based on the ST-LCM (11) with respect to the other two proposed prediction techniques.

From the comparison with respect to the kriging results, it is clear that the direct and cross-correlations of the primary and secondary variables contribute to enhance the weights of the cokriging estimator and then to improve the predictions. In addition, the comparison with respect to the use of the intrinsic coregionalization model has highlighted the positive effect of retaining four scales of variability in the construction of the final ST-LCM and in the production of better predictions. The suitability of the ST-LCM (11), confirmed by all the procedures above discussed, has allowed the adoption of this model to predict Rn flux values over the Veneto Region at unobserved time points, as detailed in the next section.

## 7. Rn Flux Prediction Maps

**Figure 7:**
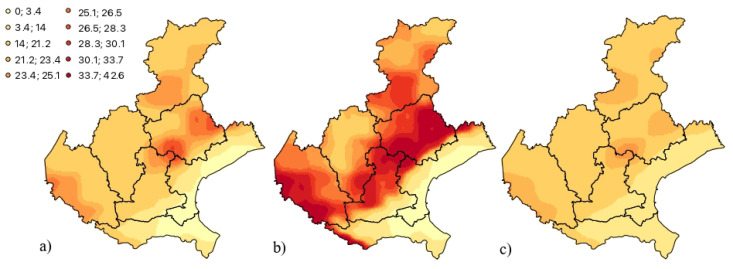
Prediction maps of Rn flux (in KBqm${}^{-2}$ s${}^{-1}$) monthly averages for (**a**) May, (**b**) August and (**c**) November 2022.

The fitted and validated model (11) has been considered to produce predictions of the Rn flux from May 2022 (the month after the last one available in the data set) up to December 2022, over the study area. For this aim, space–time cokriging has been applied, by using the GSLib routine “COK2ST” [29] whose parameter file was implemented with all required information concerning, among others, the basic components’ models and the neighbourhood of sample data to be used in the cokriging system for both the primary variable (Rn flux) and the auxiliary variables (the three meteorological variables herein analyzed). In this way, residuals of monthly Rn flux have been first predicted, then the seasonal component, previously calculated point-by-point as described in Section 5.3, has been added to predictions in order to obtain the Rn flux monthly forecasts. In Figure 7, the contour maps of the predicted Rn flux values for May, August and November 2022 are shown: such months selection is justified by the particular behavior of the Rn flux during the year with the highest values during the summer time and the lowest ones during the winter. Indeed, as highlighted in [72] and already discussed in Section 5.3, in general a temperature increase favors the flux of radon from soil to atmosphere, therefore the Rn flux tends to increase in the spring–summer season and to decrease in the winter, due to the interaction with the meteorological conditions which characterize the study area during the warmest and the coldest months of the year.

On the other hand, regarding the spatial profile, the prediction maps clearly show those territories with a very high exposure to Rn exhalation from the ground (Figure 7). In particular, the territories located in the north-eastern part of the Veneto Region, namely in the Province of Treviso and Belluno, in the center part belonging to the Province of Padua and in the eastern one of the Region over the Province of Verona. This portion of land, which crosses the study area from northeast to southwest, is characterized by complex geo-lithological conditions which may favor the exhalation of Rn gas from the soil, as was pointed out in previous research [58,60,72] that focused on the investigation of Rn outdoor and indoor concentrations over the Veneto Region.

## 8. Rn Flux Risk Maps

Risk assessment maps have been associated to the prediction maps. Indicator kriging has been applied to assess the probability that Rn flux predictions for August 2022 exceed, alternatively,

(a) the 25th percentile (19.326 KBqm${}^{2}$s${}^{-1}$),
(b) the average value (22.75 KBqm${}^{2}$s${}^{-1}$),
(c) the median (23.994 KBqm${}^{2}$s${}^{-1}$)

of the distribution of the historical data measured in August from 2006 to 2021 at all spatial points. The choice of this month has been justified by the need to investigate the risk of radon exhalation when warmer climatic conditions in the study area favor the increase of Rn flux. Thus, three threshold values $z_{1}=19.326,z_{2}=22.75$ and $z_{3}=23.994$ have been considered as the target thresholds in order to define the following indicator variables

(14) $$\begin{array}{c} I_{1}\left(s,t;z_{1}\right)=\left\{\begin{array}{cc} 1 & \;if\;Rn>z_{1},\\ \; & \\ 0 & \;\mathrm{otherwise}, \end{array}\right.\;I_{2}\left(s,t;z_{2}\right)=\left\{\begin{array}{cc} 1 & \;if\;Rn>z_{2},\\ \; & \\ 0 & \;\mathrm{otherwise}, \end{array}\right.\\ I_{3}\left(s,t;z_{3}\right)=\left\{\begin{array}{cc} 1 & \;if\;Rn>z_{3},\\ 0 & \;\mathrm{otherwise}, \end{array}\right. \end{array}$$

where s∈D,t∈T, and $z_{1},z_{2},z_{3}$ represent the thresholds above listed.

**Figure 8:**
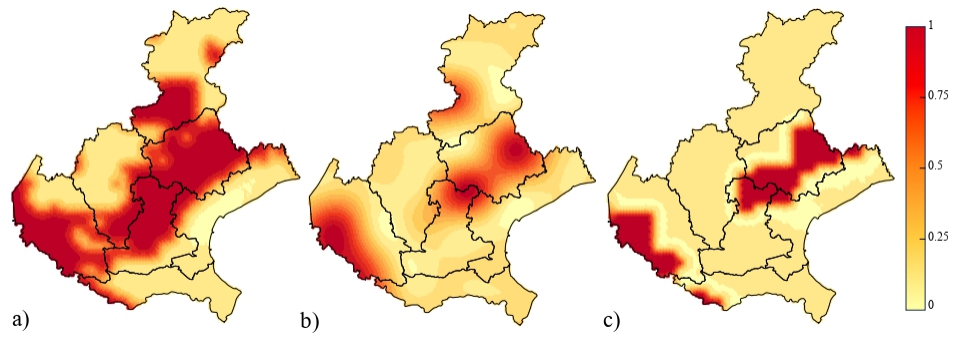
Risk maps of the probability that Rn flux predicted in August 2022 exceeds (**a**) the 25th percentile ($z_{1}=19.326$ KBqm${}^{2}$s${}^{-1}$), (**b**) the mean ($z_{2}=22.75$ KBqm${}^{2}$s${}^{-1}$), (**c**) the median value ($z_{3}=23.994$ KBqm${}^{2}$s${}^{-1}$) of the corresponding historical measurements.

Successively, three spatio-temporal indicator kriging procedures were conducted to obtain the risk maps (Figure 8), referred to August 2022, of the exceeding the chosen Rn flux threshold levels over the study area [73]. Note that these probability maps have been produced by considering only the variable Rn flux, taking into account that the kriging performance in Table 3 was satisfactory. However, the multivariate indicator approach might be also used in order to include the behavior of the meteorological conditions.

All maps have highlighted the presence of hazardous areas in the south-western part of the Veneto Region over the Province of Verona, as well as in the north-eastern sub-area inside the Province of Treviso, where there is a very high probability level that even the largest fixed threshold (the median) is overcome. Apart from the map in Figure 8a constructed for a moderate threshold value, it is important to highlight that the risk areas emerging in the last two maps (red territories in Figure 8b,c) include several districts that are among the most populated ones in the region. Therefore, any possible plans of urban expansion will require an accurate evaluation taking into account the high risk of Rn gas exhalation from the soil.

In conclusion, the obtained probability maps represent very useful tools for detecting areas in need of strong controls, especially under the consideration that Rn flux measurements could be a fine proxy of outdoor and indoor Rn concentrations which have been proved to be highly dangerous to human health.

## 9. Summary

In the present paper, a critical review of the tools of the multivariate geostatistics for spatio-temporal modeling and predictions was provided. In addition, the ST-LCM fitting procedure, based on the joint diagonalization of the sample covariance matrices computed at different lags, was applied to describe the spatio-temporal correlation among Rn flux and some meteo-climatic monthly conditions (mean air temperature, minimum humidity and evapotranspiration) measured over the Veneto Region from 2006 to 2022. After model validation, a comparative analysis of the prediction performances obtained from the use of cokriging based on an alternative multivariate model and of kriging based only on the product–sum fitted exclusively for the Rn flux, confirmed the superiority of the ST-LCM. Thus, the proposed analysis pointed out that the efforts required for the identification of the multivariate spatio-temporal correlation model are crucial for the final results and are rewarded with reliable estimations.

In conclusion, the results obtained in this paper can be considered particularly important since they might contribute to an accurate assessment of indoor radon levels, as well as to the identification of radon priority areas. These might then be used by local or regional authorities for land-use planning and urban development. In the future, other multivariate approaches can be used for comparative purposes [74].

## Figures and Tables

**Table 1:** Covariance model parameters estimated for basic components in (12).

l=1	l=2	l=3	l=4
$k_{1_{1}}=0.0047$	$k_{1_{2}}=15.0756$	$k_{1_{3}}=15.1367$	$k_{1_{4}}=0.01$
$k_{2_{1}}=0.0358$	$k_{2_{2}}=10.6321$	$k_{2_{3}}=30.703$	$k_{2_{4}}=17.0644$
$k_{3_{1}}=0.0081$	$k_{3_{2}}=33.4605$	$k_{3_{3}}=0.01$	$k_{3_{4}}=2.9732$
$a_{1}$=20 km	$a_{2}$=30 km	$a_{3}$=55 km	$a_{4}$=120 km
$b_{1}$=2 months	$b_{2}$=3 months	$b_{3}$=7 months	$b_{4}$=12 months

**Table 2:** Statistics for models’ performance assessment.

	ST-LCM with 4 Basic Models		ST-LCM with 1 Basic Model	
	RAE	RMAE	RAE	RMAE
Rn flux	0.156	0.152	0.839	1.018
Rn flux vs. $T_{M}$	0.623	0.544	1.280	1.419
Rn flux vs. $H_{m}$	0.454	0.426	0.422	0.377
Rn flux vs. $ET_{0}$	0.697	0.717	1.353	1.588
$T_{M}$	0.304	0.223	0.641	0.637
$T_{M}$ vs. $H_{m}$	0.392	0.378	0.851	0.825
$T_{M}$ vs. $ET_{0}$	0.495	0.497	0.154	0.130
$H_{m}$	0.857	0.886	1.572	2.219
$H_{m}$ vs. $ET_{0}$	0.916	0.898	2.249	2.377
$ET_{0}$	0.969	0.748	2.110	2.302

**Table 3:** Statistics of the performances of prediction methods.

	Cokriging with ST-LCM in (11)	Cokriging with ST-LCM in (13)	Kriging
Correlation coefficient	0.909 *	0.792 *	0.787 *
RAE	0.222	0.311	0.325
RMAE	0.124	0.246	0.145

* Correlation is significant at 1% level.

## Data Availability

Publicly available datasets were analyzed in this study. Rn flux data are available at https://meta.icos-cp.eu/objects/5-Z-zRaqFgddALv0ohLonzWD. Raw data on $T_{M}$, $H_{m}$ and $ET_{0}$ are available at https://www.arpa.veneto.it.
